# From Images to Specimens: The Impact of Tactile, Three-Dimensional Learning in Dental Anatomy

**DOI:** 10.3390/dj14040245

**Published:** 2026-04-21

**Authors:** Anna Tostrup Kristensen, Noora Helene Thune, Hugo Lewi Hammer, Qalbi Khan, Tor Paaske Utheim, Camilla Sofia Miranda Kristoffersen, Camilla Elise Øxnevad Ziesler, Amer Sehic

**Affiliations:** 1 Institute of Oral Biology, Faculty of Dentistry, University of Oslo, 0371 Oslo, Norway; annatk@student.odont.uio.no (A.T.K.); nooraht@student.odont.uio.no (N.H.T.); qalbi.khan@odont.uio.no (Q.K.); uxutto@ous-hf.no (T.P.U.); cskristo@student.odont.uio.no (C.S.M.K.); ceziesle@student.odont.uio.no (C.E.Ø.Z.); 2 Department of Medical Biochemistry, Oslo University Hospital, 0371 Oslo, Norway; 3 Department of Plastic and Reconstructive Surgery, Oslo University Hospital, 0424 Oslo, Norway; hugoh@oslomet.no; 4 Department of Computer Science, Faculty of Technology, Art and Design, Oslo Metropolitan University, 0176 Oslo, Norway; 5 Department of Public Health and Sport Sciences, Inland Norway University of Applied Sciences, 2418 Elverum, Norway

**Keywords:** dental anatomy, oral macroscopic anatomy, knowledge retention, tactile learning, three-dimensional specimens

## Abstract

**Background:** A solid understanding of dental and craniofacial anatomy is essential for reliable clinical practice, yet long-term retention of anatomical knowledge is known to decline as students progress through their training. Although digital- and image-based resources are widely used in anatomy education, it remains unclear whether two-dimensional (2D) representations support durable recognition of complex anatomical structures. This study investigated whether tactile engagement with real three-dimensional (3D) anatomical specimens enhances long-term identification accuracy compared with standardized 2D images. **Materials and Methods:** Eighty-nine fifth-year dental students at the University of Oslo were assessed approximately 2.5 years after completing their formal anatomy course. All students completed two sequential identification tests on the same day: a 2D image-based test followed immediately by an equivalent test using real extracted human teeth and real skull bones. The assessments comprised 28 osteology structures and 14 teeth. Performance between conditions was compared using mixed-effects logistic regression with test modality as a fixed effect and participant and anatomical item as crossed random effects. **Results:** Overall identification accuracy increased from 52% in the 2D condition to 74% in the 3D tactile condition (*p* < 0.0001). Mean accuracy for osteology improved from 0.519 (SD = 0.074) to 0.708 (SD = 0.072) (*p* < 0.0001), while tooth morphology scores increased from 0.535 (SD = 0.097) to 0.795 (SD = 0.110) (*p* < 0.0001). All individual teeth and nearly all osteological structures showed significantly higher identification accuracy in the 3D condition. The largest gains were observed for structures with low 2D recognition. **Conclusions:** Tactile interaction with real 3D anatomical specimens substantially enhances long-term recognition of dental and craniofacial anatomy. These findings support the continued integration of hands-on, three-dimensional learning alongside digital resources in dental anatomy education to promote durable anatomical understanding and clinical preparedness.

## 1. Introduction

A solid understanding of anatomy is fundamental to safe and effective clinical practice [1,2,3]. In dentistry, thorough knowledge of oral macroscopic anatomy underpins diagnostic accuracy, surgical procedures, restorative treatment, and the management of clinical complications [4,5]. Insufficient anatomical understanding may lead to technical errors and compromise patient safety, highlighting the critical role of effective anatomy education in undergraduate dental training [3]. The capacity to recognize and mentally reconstruct three-dimensional anatomical structures is essential not only for operative dentistry and oral surgery, but also for radiographic interpretation, endodontic treatment, and complication management. When anatomical knowledge becomes increasingly detached from its physical and spatial context during training, its applicability and effectiveness in clinical practice may be diminished [5,6]. Accordingly, effective dental treatment depends on accurate three-dimensional anatomical understanding to ensure stable occlusion, coordinated mandibular movements, and temporomandibular joint integrity, increasingly supported by three-dimensional planning and emerging AI-based tools.

Traditionally, anatomy education in dentistry has relied on a combination of lectures, atlases, different digital resources, dissections, and direct examination of real human material, including skulls, individual bones, wet specimens, and extracted teeth [7,8,9,10]. These approaches provide students with multisensory learning experiences, allowing them to see, touch, and manipulate anatomical structures in three dimensions [9,11]. Educational and cognitive theories suggest that such multisensory engagement strengthens memory encoding and supports spatial understanding [11,12]. In the context of dentistry, tactile learning is particularly relevant, as clinical practice depends heavily on fine motor control, spatial orientation, and the ability to interpret subtle tactile cues during procedures [4,13]. Despite its central role in clinical competence, long-term retention of anatomical knowledge remains a persistent challenge for dental students as they progress through increasingly practice-oriented phases of their education [14,15,16]. Several studies have demonstrated that recall and accurate identification of anatomical structures decline over time, even for concepts that are critical to routine clinical decision-making [6,17,18].

In recent years, digital and image-based learning resources have become increasingly prominent in anatomy education. High-resolution 2D images, digital atlases, and interactive platforms offer accessibility, flexibility, and repeated exposure to anatomical content. These tools align well with contemporary learning habits and have clear pedagogical value, particularly for introductory learning and revision [19,20,21,22]. However, it remains uncertain whether visual exposure alone is sufficient to support durable anatomical knowledge, especially for complex structures with irregular shapes, deep anatomical locations, or subtle morphological differences. While digital tools may enhance conceptual understanding, their ability to sustain long-term recognition of real anatomical structures has been questioned [23,24,25,26].

The present study addresses this gap by directly comparing dental students’ long-term ability to identify teeth and craniofacial structures using two different modalities: standardized 2D images and real three-dimensional anatomical specimens. Fifth-year dental students at the University of Oslo were assessed approximately 2.5 years after completing their formal anatomy instruction, providing insight into long-term knowledge retention rather than short-term learning effects. By using a paired within-subject design, the study aimed to test whether tactile interaction with real extracted teeth and skull bones results in significantly higher identification accuracy than interpretation of 2D images, and to determine whether this effect is consistent across different anatomical regions and levels of item difficulty.

## 2. Materials and Methods

### 2.1. Teaching Context and Anatomical Background

At the Faculty of Dentistry (UiO), oral macroscopic anatomy is taught during the second year of the five-year dental program. The anatomy component forms part of a broader module in craniofacial structure and function and extends over approximately six weeks. Teaching includes a combination of lectures, anatomical models, real human skulls, individual bones, and dissected wet specimens. During practical sessions, students are encouraged to handle, rotate, and examine real specimens to deepen three-dimensional understanding and develop tactile familiarity with anatomical structures. This exposure includes both tooth morphology and craniofacial osteology. Students also use supplementary 2D visual materials, such as atlases, digital images, and structured diagrams. Formal assessment at the end of the second year includes a written exam and a practical station-based examination requiring identification of structures on both 2D images and real specimens.

Following completion of the second-year anatomy course, students progress to clinical disciplines where anatomical knowledge is applied in patient treatment. Approximately 2.5 years later, in the fifth year of the program, students participate in a short dissection course intended to reinforce vertical integration and refresh their understanding of relevant anatomical structures. The present study was conducted immediately prior to this fifth-year dissection course.

### 2.2. Study Design and Testing Procedure

**Figure 1:**
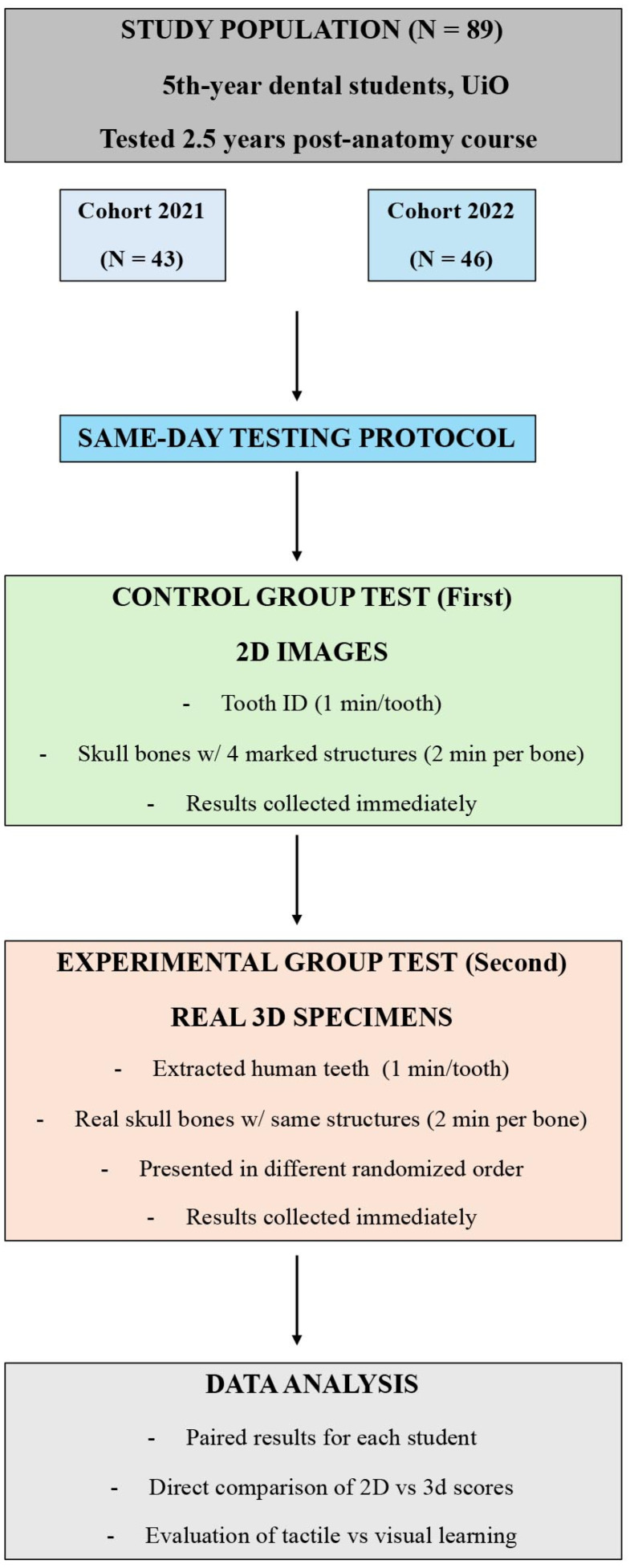
**Flowchart of the study design and testing procedure.** Fifth-year dental students at the Faculty of Dentistry, UiO (University of Oslo), were assessed approximately 2.5 years after completing their anatomy course. Two cohorts participated: 43 students in 2021 and 46 students in 2022 (total *n* = 89). In both years, all students completed two tests on the same day. First, the Control Group Test consisted of identifying human teeth and marked anatomical structures on skull bones using standardized 2D images (1 min per tooth; 2 min per bone). Immediately afterward, students completed the Experimental Group Test, which used the same teeth and skull bones presented as real 3D anatomical specimens in a newly randomized order. Test duration was identical (1 min per tooth; 2 min per bone). Results from both sessions were collected directly after each test, and paired for each student, enabling direct within-subject comparison of performance on 2D images versus real anatomical specimens.

The study included two cohorts of fifth-year dental students: 43 students in 2021 and 46 students in 2022, comprising a total of 89 participants (Figure 1). The number of participants was limited to the available fifth-year student cohorts during the study period, as inclusion required prior completion of the same standardized anatomy curriculum and participation immediately before the scheduled dissection course. All students had received the same foundational anatomy teaching during their second year, and none had been exposed to additional formal anatomy instruction in the intervening years. The study design involved two sequential tests conducted on the same day: a 2D image-based test (Control Group Test) followed immediately by a real specimen test (Experimental Group Test). Each student completed both tests, enabling paired within-subject analysis (Figure 1). Informed consent was obtained from all participating students prior to participation.

**Figure 2:**
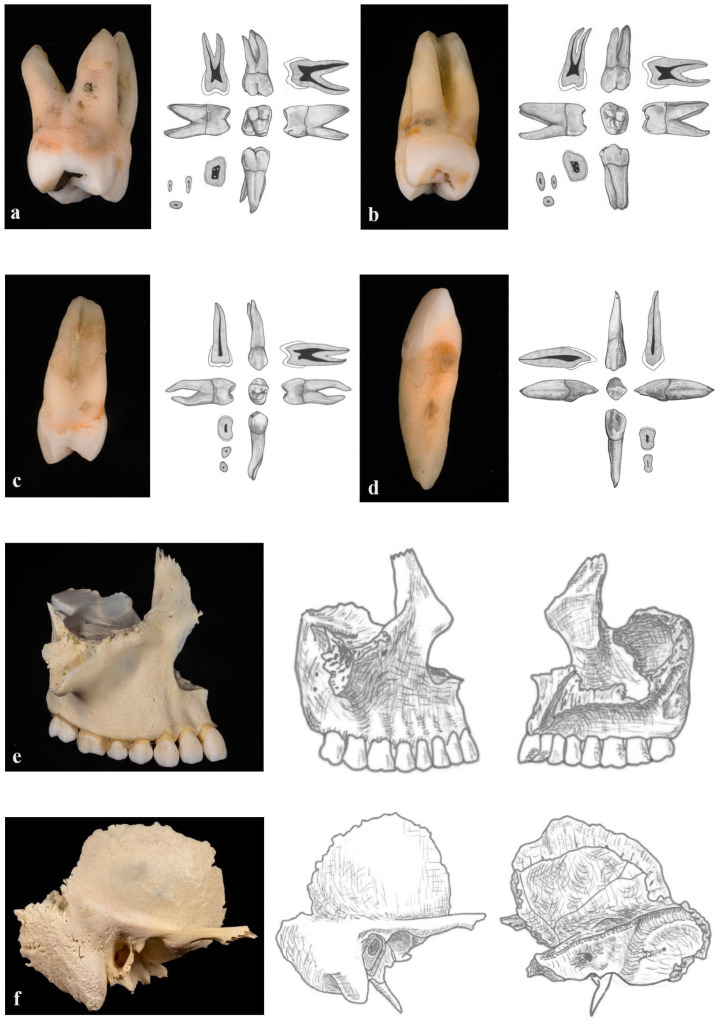
**Examples of anatomical specimens and corresponding 2D images used in the study.** Panels (**a**–**d**) show representative teeth included in the assessments. In each panel, the left image displays a real extracted human tooth (3D specimen), and the right images show the corresponding 2D illustrations of the same tooth viewed from multiple aspects. Panels (**e**,**f**) illustrate the skull bones used for the bone identification tasks. In each case, the left image shows the real human bone specimen (lateral view only), while the right images present 2D illustrations of the same bone from both lateral and medial aspects. Panel E displays the maxilla, and panel F shows the temporal bone. These examples demonstrate the visual differences between tactile 3D specimens and their corresponding 2D representations used in the Control and Experimental test conditions.

In the first test session, students were presented with standardized 2D images of human teeth and craniofacial bones (Figure 2). For the tooth identification component, each student was given one minute per tooth to identify the correct tooth type. For the osteology component, students were shown images of skull bones with four anatomical structures marked. They were allotted two minutes per bone to identify all marked structures. All responses were recorded on individual answer sheets, which were collected immediately after completion of the test.

Immediately following the 2D assessment, students moved to a separate room for the second test session. Here, they were presented with the same set of anatomical items, extracted human teeth and human skull bones, as real 3D specimens rather than images (Figure 2). The teeth and bones were presented in a newly randomized order to reduce recall bias from the first test. Students were given the same amount of time as in the 2D test: one minute per tooth and two minutes per bone. Upon completion, answer sheets from the real-specimen test were collected immediately.

The same procedure was used for both cohorts, and the materials used were identical across years. For each participant, results from the 2D and 3D tests were paired, allowing direct comparison of performance under the two conditions. This design enabled evaluation of the impact of tactile engagement with real anatomical structures on long-term recognition accuracy 2.5 years after formal instruction.

### 2.3. Statistical Analysis

To evaluate differences in performance between the 2D and 3D modalities, mixed-effects logistic regression models were employed. The models included type of task (2D vs. 3D) as a fixed effect, while Participant ID and Task were entered as crossed random effects. The Participant ID random intercept accounted for repeated measures and baseline differences in student ability (i.e., some students performing consistently better than others). The task random intercept accounted for variations in item difficulty (i.e., some anatomical structures being harder to identify than others).

## 3. Results

### 3.1. Overall Test Performance

All 89 students completed both test conditions, each comprising identification of 42 anatomical items (28 osteological structures and 14 teeth) (Table 1 and Table 2). Marked differences in performance were observed between the two modalities. In the 2D image-based test, students achieved 1961 correct responses out of a possible 3738, corresponding to an overall accuracy of approximately 52%. When the same anatomical items were assessed using real specimens, the number of correct responses increased to 2754, yielding an accuracy of approximately 74%. At the individual level, students correctly identified a mean of 22.0 out of 42 items in the 2D condition, compared with 30.9 out of 42 in the 3D tactile condition. Improvement was observed for all 42 anatomical structures when assessed in the 3D condition, with the largest gains seen for items that exhibited low recognition rates in the 2D image-based test.

Mixed-effects logistic regression analysis demonstrated a highly significant overall effect of test modality, with substantially higher odds of correct identification in the 3D condition compared with the 2D condition (*p* < 0.0001). This effect remained robust after accounting for repeated measurements within students and differences in item difficulty.

When osteology and tooth morphology were analyzed separately, the same pattern was observed. For osteology, mean accuracy increased from 0.519 (SD = 0.074) in the 2D condition to 0.708 (SD = 0.072) in the 3D condition (*p* < 0.0001). For tooth morphology, mean accuracy increased from 0.535 (SD = 0.097) to 0.795 (SD = 0.110) (*p* < 0.0001). Thus, tactile engagement with real specimens was associated with significantly improved long-term recognition across both anatomical domains.

**Table 1:** **Tooth morphology identification performance in 2D and 3D conditions.** Mean identification scores (proportion correct) and standard deviations (SD) for individual teeth assessed using 2D image-based testing and 3D testing with real extracted human teeth in fifth-year dental students (*n* = 89). Scores are shown for total tooth morphology and for each tooth separately. Differences between conditions were analyzed using mixed-effects logistic regression with test modality (2D vs. 3D) as a fixed effect and Participant ID and tooth as crossed random effects. *p*-values indicate the statistical significance of differences between 2D and 3D conditions.

Teeth	Mean Score 2D (SD)	Mean Score 3D (SD)	*p*-Value
**Tooth morphology (total)**	0.535 (0.097)	0.795 (0.110)	<0.0001
**Tooth 11**	0.730 (0.446)	0.910 (0.288)	<0.0001
**Tooth 22**	0.674 (0.471)	0.955 (0.208)	<0.0001
**Tooth 41**	0.169 (0.376)	0.719 (0.452)	<0.0001
**Tooth 13**	0.427 (0.497)	0.809 (0.395)	<0.0001
**Tooth 43**	0.506 (0.503)	0.674 (0.471)	0.0034
**Tooth 34**	0.618 (0.489)	0.798 (0.404)	<0.0001
**Tooth 25**	0.573 (0.497)	0.787 (0.412)	<0.0001
**Tooth 14**	0.697 (0.462)	0.843 (0.366)	<0.0001
**Tooth 36**	0.787 (0.412)	0.955 (0.208)	<0.0001
**Tooth 48**	0.281 (0.452)	0.607 (0.491)	<0.0001
**Tooth 16**	0.775 (0.420)	0.910 (0.288)	<0.0001
**Tooth 27**	0.652 (0.479)	0.798 (0.404)	<0.0001
**Tooth 54**	0.292 (0.457)	0.674 (0.471)	<0.0001
**Tooth 85**	0.315 (0.467)	0.685 (0.467)	<0.0001

### 3.2. Tooth Identification

Identification accuracy for individual teeth increased consistently when students examined real extracted teeth (Table 1). In the 2D image-based test, correct identification rates varied substantially across teeth, with mean scores ranging from 0.169 (SD = 0.376) for tooth 41 to 0.787 (SD = 0.412) for tooth 36. Anterior maxillary teeth and selected posterior permanent teeth (e.g., teeth 11, 16, and 36) demonstrated relatively high recognition in the 2D condition, whereas mandibular incisors, third molars, and deciduous teeth exhibited low accuracy.

In the 3D tactile condition, identification accuracy increased significantly for every tooth examined (all *p* < 0.0001). Particularly large gains were observed for teeth that were difficult in the 2D condition. For example, mean scores for tooth 41 increased from 0.169 to 0.719, tooth 48 from 0.281 to 0.607, tooth 54 from 0.292 to 0.674, and tooth 85 from 0.315 to 0.685. In contrast, teeth that were already relatively well recognized in 2D, such as teeth 11 and 36, also showed significant but smaller absolute improvements, reaching mean scores of 0.910 and 0.955, respectively, in the 3D condition. Overall, assessment using real teeth resulted in higher identification accuracy across all tooth types and reduced the performance gap between teeth that were easy and difficult to identify in the 2D condition, although some inter-tooth variability remained.

**Table 2:** **Osteology identification performance in 2D and 3D conditions.** Mean identification scores (proportion correct) and standard deviations (SD) for craniofacial osteology structures assessed using 2D image-based testing and 3D testing with real skull bones in fifth-year dental students (*n* = 89). Scores are presented for anatomical regions and individual structures. Differences between conditions were analyzed using mixed-effects logistic regression with test modality (2D vs. 3D) as a fixed effect and Participant ID and anatomical item as crossed random effects. *p*-values indicate the statistical significance of differences between 2D and 3D conditions.

Bones and Anatomical Structures	Mean Score 2D (SD)	Mean Score 3D (SD)	*p*-Value
**Osteology (total)**	0.519 (0.074)	0.708 (0.072)	<0.0001
**Mandible**	0.649 (0.195)	0.789 (0.180)	<0.0001
mandibular foramen	0.876 (0.331)	0.944 (0.232)	0.0005
head of the mandible	0.809 (0.395)	0.865 (0.343)	<0.0001
pterygoid tuberosity	0.382 (0.489)	0.652 (0.479)	0.0086
pterygoid fovea	0.528 (0.502)	0.697 (0.462)	<0.0001
**Maxilla**	0.713 (0.175)	0.848 (0.163)	<0.0001
canine fossa	0.663 (0.475)	0.820 (0.386)	<0.0001
maxillary tuberosity	0.831 (0.376)	0.899 (0.303)	<0.0001
infraorbital groove	0.472 (0.502)	0.764 (0.427)	<0.0001
incisive canal	0.888 (0.318)	0.910 (0.288)	<0.0001
**Sphenoid bone**	0.475 (0.210)	0.683 (0.212)	<0.0001
carotid groove	0.393 (0.491)	0.573 (0.497)	<0.0001
foramen ovale	0.607 (0.491)	0.775 (0.420)	<0.0001
superior orbital fissure	0.326 (0.471)	0.652 (0.479)	<0.0001
pterygoid hamulus	0.573 (0.497)	0.730 (0.446)	<0.0001
**Temporal bone**	0.402 (0.198)	0.632 (0.181)	<0.0001
articular tubercle	0.506 (0.503)	0.719 (0.452)	0.0702
internal acoustic meatus	0.483 (0.503)	0.719 (0.452)	0.0073
mastoid process	0.438 (0.499)	0.584 (0.496)	0.0001
trigeminal impression	0.180 (0.386)	0.506 (0.503)	<0.0001
**Frontal bone**	0.407 (0.182)	0.567 (0.176)	<0.0001
superciliary arch	0.315 (0.467)	0.461 (0.501)	<0.0001
zygomatic process	0.404 (0.494)	0.618 (0.489)	0.0024
nasal spine	0.472 (0.502)	0.562 (0.499)	0.0432
lacrimal gland fossa	0.438 (0.499)	0.629 (0.486)	0.0028
**Occipital bone**	0.581 (0.172)	0.767 (0.176)	<0.0001
external occipital protuberance	0.404 (0.494)	0.674 (0.471)	0.0100
occipital condyle	0.483 (0.503)	0.674 (0.471)	0.0036
foramen magnum	0.764 (0.427)	0.888 (0.318)	<0.0001
hypoglossal canal	0.674 (0.471)	0.831 (0.376)	<0.0001
**Whole cranium**	0.407 (0.204)	0.669 (0.212)	<0.0001
greater palatine foramen	0.719 (0.452)	0.921 (0.271)	<0.0001
mastoid notch	0.124 (0.331)	0.472 (0.502)	<0.0001
carotid canal	0.438 (0.499)	0.663 (0.475)	0.0030
foramen spinosum	0.348 (0.479)	0.618 (0.489)	0.0086

### 3.3. Osteology

A similar pattern was observed for craniofacial osteology (Table 2). On the 2D image-based test, mean identification accuracy varied considerably across structures and anatomical regions. Structures located in the cranial base and deep anatomical spaces were particularly challenging, with very low mean scores for items such as the mastoid notch (0.124), trigeminal impression (0.180), superior orbital fissure (0.326), and foramen spinosum (0.348).

When the same structures were assessed using real skull bones, identification accuracy increased significantly for nearly all osteology items. At the regional level, mean scores improved for the mandible, maxilla, sphenoid, temporal, frontal, occipital bones, and the whole cranium (all *p* < 0.0001). The largest absolute gains were observed for structures that were poorly recognized on 2D images. For example, mean scores for the mastoid notch increased from 0.124 to 0.472, the trigeminal impression from 0.180 to 0.506, the superior orbital fissure from 0.326 to 0.652, and the infraorbital groove from 0.472 to 0.764 (all *p* < 0.0001).

Even for structures with relatively high recognition rates in the 2D condition, such as the incisive canal, foramen magnum, hypoglossal canal, and greater palatine foramen, identification accuracy increased further when assessed in the 3D tactile condition. Most individual osteological items demonstrated statistically significant modality effects, with the articular tubercle as the only exception, where the difference between conditions did not reach statistical significance (*p* = 0.0702). Tactile examination improved long-term identification of craniofacial structures and reduced the number of items with very low success rates; however, several anatomically complex structures remained difficult to identify even when real specimens were used, indicating persistent challenges despite tactile access.

## 4. Discussion

The present study investigated long-term retention of dental and craniofacial anatomy by comparing students’ ability to identify anatomical structures using 2D images and real three-dimensional (3D) anatomical specimens. Assessments were conducted approximately 2.5 years after formal anatomy instruction, providing insight into durable learning rather than short-term recall. The results demonstrate a clear and consistent advantage of tactile, 3D interaction with real anatomical material. Across all anatomical domains examined, students demonstrated significantly higher accuracy in structure identification when handling real specimens compared with interpreting standardized 2D images.

The magnitude of this difference is noteworthy. Overall identification accuracy increased from approximately 52% in the 2D condition to 74% in the 3D condition, with statistically significant improvements observed for nearly all individual items. This pattern was evident for both tooth morphology and craniofacial osteology, indicating that the benefit of tactile engagement is not limited to a specific anatomical category. These findings align with theories of multisensory learning, which posit that information encoded through multiple sensory channels, particularly vision and touch, yields more robust and retrievable memory representations [27]. In anatomy education, tactile input provides access to spatial features such as depth, curvature, orientation, and surface texture, which are difficult to fully convey through flat images [12,27].

Importantly, the largest gains were observed for structures that were poorly recognized in the 2D condition, including mandibular incisors, deciduous molars, and deep or complex cranial base structures such as the mastoid notch, superior orbital fissure, trigeminal impression, and infraorbital groove. These teeth and osteological structures have previously been shown to be among the most difficult for students to identify and recall, even when assessed using real teeth and skeletal specimens [9,10,28,29]. These anatomical features are characterized by subtle morphology, variable orientation, or limited visibility in 2D representations. The ability to rotate and physically explore the specimen appears to substantially reduce cognitive load and support more accurate identification. This suggests that tactile engagement is particularly valuable for structures that challenge purely visual interpretation [30].

At the same time, the tactile condition also improved performance for structures that were already relatively well recognized in 2D. This indicates that tactile inspection does not merely compensate for weak visual recognition but may also consolidate and refine existing anatomical knowledge. Handling real specimens likely reinforces spatial relationships and contextual understanding, helping students resolve uncertainty and strengthen confidence in their identification [12]. Such reinforcement is highly relevant in dentistry, where clinical competence relies on accurate mental reconstruction of three-dimensional anatomy during procedures that depend heavily on tactile feedback. Evidence from studies on haptic feedback devices in preclinical dental training show that realistic tactile sensations can help students develop motor skills, hand-eye coordination and understanding of oral anatomy, supporting the broader principle that multisensory engagement contributes to consolidating anatomical knowledge [31].

The timing of the assessment offers important insight into knowledge retention. By the fifth year, students had extensive clinical experience but had not received formal anatomy instruction for more than two years. Weaker performance in the 2D condition suggests that image-based assessment may be less effective in supporting the recall of anatomical knowledge over time, despite its clinical relevance. In contrast, the marked improvement observed when students interacted with real specimens implies that some anatomical knowledge remains accessible but is more readily retrieved when supported by tactile and three-dimensional input. This finding points to the potential role of tactile learning in re-activating and stabilizing anatomical knowledge that might otherwise appear lost when assessed using abstract or image-based formats alone. This aligns with a growing body of research showing that three-dimensional physical models yield superior retrieval of spatial and anatomical information than two-dimensional [32]. This advantage likely arises because tactile learning is inherently active, engaging somatosensory systems, unlike visual or auditory information, which may be more passively received. Active learning research confirms that this physical engagement enhances outcomes as students learn concepts while directly experiencing them [12,33].

These results have implications for both curriculum design and assessment practices. The widespread use of digital and image-based resources in anatomy education offers clear advantages in terms of accessibility and standardization [20,34]. However, the present findings suggest that reliance on 2D materials alone may be insufficient to support long-term retention of complex anatomical knowledge [35]. For a profession that depends on three-dimensional reasoning and tactile discrimination, continued exposure to real anatomical material, or high-fidelity physical equivalents, appears essential. Rather than viewing digital tools and tactile learning as competing approaches, the data support a complementary model in which visual resources introduce and reinforce concepts, while hands-on experience anchors them in durable spatial understanding [30,35]. Furthermore, artificial intelligence and digital innovations are increasingly integrated into modern dentistry and education, offering potential for improved diagnostics, treatment planning, and accessibility. However, while these developments suggest real transformation, they must be complemented by active, hands-on learning to avoid superficial understanding and to preserve essential practical and tactile skills required for clinical competence.

Some limitations should be considered when interpreting these findings. First, the study employed a fixed test order, with the 2D image-based assessment always preceding the 3D specimen-based assessment. This design raises the possibility of a short-term learning or priming effect, whereby exposure to anatomical structures in the 2D test could improve subsequent performance in the 3D test. Although such an effect cannot be entirely excluded, several factors suggest that it is unlikely to account for the magnitude and consistency of the observed differences. Despite these considerations, a learning or priming effect from repeated exposure to the same structures on the same day cannot be excluded. The assessments were conducted under time pressure, focused on identification rather than instruction, and involved many distinct items. Moreover, the order of anatomical items was newly randomized between the two tests, reducing direct recall of specific answers. Importantly, the improvements were largest for structures that were poorly recognized in 2D, which argues against a simple practice effect and instead supports a genuine advantage of tactile, three-dimensional interaction. Nonetheless, future studies could strengthen causal inference by counterbalancing test order across participants. Additional limitations include the single-institution design and the absence of intermediate reinforcement data between the second and fifth years. In addition, informal reinforcement of anatomical knowledge through clinical exposure during the intervening years may have contributed to students’ performance, even in the absence of formal anatomy teaching. While the within-subject design strengthens internal validity, replication across institutions and curricula would enhance generalizability. Finally, although real anatomical specimens represent a pedagogical gold standard, their availability is becoming more limited, and future work should explore whether high-fidelity physical or digital 3D models can reproduce similar benefits.

## 5. Conclusions

Our findings underscore the educational value of multisensory, three-dimensional learning in dental anatomy and highlight the limitations of image-based assessment as a sole measure of anatomical competence. While digital and 2D resources remain important for accessibility and conceptual learning, they do not fully substitute for the perceptual richness and spatial understanding afforded by real anatomical material. Integrating tactile, three-dimensional experiences throughout the dental curriculum, both during initial instruction and as part of later reinforcement, may support more durable anatomical knowledge and better align educational practices with the clinical demands of dentistry.

## Data Availability

The original contributions presented in the study are included in the article, further inquiries can be directed to the corresponding author.
